# The Intercepting Trap on Its Trial

**Published:** 1908-12-05

**Authors:** 


### PUBLIC HEALTH AND HYGIENE.

### THE INTERCEPTING TRAP ON ITS TRIAL.

THE President of the Local Government Board has appointed a Departmental Committee to inquire into the merits of the intercepting trap. To those members of the medical profession who are not directly concerned in sanitary administration this may seem an inquiry of small moment; but those whose interest in sanitary matters is more intimate will feel assured that an important issue is being reopened. The association of "drains" and certain zymotic diseases in the public, and largely in the professional, mind also, is so close that it is hardly possible to think of diphtheria, for instance, without there being called up in the mind a suggestion of defective sanitary arrangements.

Enormous sums of money have been spent during the last fifty years in perfecting the drainage and sanitary conveniences of houses throughout the country. The installation of what is known as the intercepting trap has been insisted upon by the Local Government Board, and, through them, by the local authorities as a matter of necessary and compulsory routine in drainage construction.

The ostensible object of the intercepter, it may be explained, is to disconnect the house-drainage system from that of the public sewers. This is supposed to be rendered necessary by the fact that the sewers are the common channels by which typhoid and diphtheria bacilli, and other infectious material voided by patients suffering from infectious diseases are necessarily conveyed. It is further urged that the atmosphere of the sewers, in addition to being charged with infectious germs, is also of necessity much fouler and more offensive than that of the house drains. By means of a trap situated in the course of the house drain as near to the sewer as practicable, the continuity of the atmosphere of the house drain with that of the sewer is interrupted. The effect of the mere insertion of a trap in the course of the drain is to prevent the natural ventilation of both drains and sewers. To obviate this drawback, so far as the drains are concerned, an opening is made communicating with the drain near, and on the house side of, the trap, and brought to the surface of the ground in the forecourt of the houses. This opening is known as the inlet ventilator of the drain, fresh air being supposed to enter here, and, passing along the course of the drain and up the ventilating shaft at the back, to escape above the roof.

Further complications have been introduced, rendered necessary by the fact that access to the drain must be provided in order to remove blockages, etc. These take the form of manholes on the house side of the intercepter, and a raking arm connecting the manhole in front of the house and the drain beyond the intercepter, and usually sealed by a removable stopper.

The intention of these appliances is good; but it had been asserted that in practice they are responsible for much insanitariness, and even danger to health. It is urged that the intercepter deprives the sewers of their natural ventilators—namely, the house drains and soil pipes, in consequence of which the atmosphere of the sewers becomes dangerous; that the intercepting trap is forced with the varying pressures to which inadequately ventilated sewers are necessarily subject, and that when this happens volumes of sewer air, dangerously concentrated owing to lack of ventilation, escape not only into the house drains, but through the fresh-air inlet immediately beneath the windows of the houses.

Not only are the intercepting traps forced under these circumstances, but the loose stopper of the raking arm is frequently displaced, and permanent communication is then established between the house drains and the sewers. Those who urge the discontinuance of the use of the intercepting trap say that under existing conditions this is most serious, because the accident, while insufficient adequately to ventilate the sewers, permits of the sewer air escaping at the fresh air inlet to the drains in dangerous proximity to the houses. This fresh air inlet should not be permitted, and would be quite unnecessary but for the provision of the intercepter. In addition to this the trap itself is the cause of frequent blocking of the drain. When this happens, not only do the drains fail of their primary intention, but the sewage accumulates in the manhole, which is thus converted into a cesspool in close proximity to the house. The manholes are rarely water-tight, and the sewage percolates into the soil, polluting the site of the house, and the fœtid gases given off in the course of its decomposition find a free vent at the fresh air inlet to the drain. In a paper read before the Royal Sanitary Institute in February 1906, Dr. Butler, the medical officer of health for Willesden, gave the results of a very large number of inspections of manholes he had made, amounting in all to 6,745. "In no single instance," he says, "was a drain found to be choked except at the intercepter, but no fewer than 288, or 4½ per cent. of all those inspected, were discovered to be stopped at this point. In 118 of these cases the manholes were filled with fœtid sewage, emitting its foul vapours through the drain inlet ventilator, close to the doors and windows of the houses, into which they were duly aspirated. In the 170 remaining cases where the drain was blocked, the manhole remained free of sewage, because of another accident of the system, the unstopping of the raking arm which permitted the escape of the drain contents to the sewer, and, incidentally, of the sewer gas through the manhole and drain inlet ventilator. In 654 cases this accident was observed to have occurred. That is to say, in nearly 10 per cent. of the drains examined, the intercepter, apart from its incidental drawbacks, failed absolutely of its object, the drains in these cases being in direct communication with the sewers, the sewers being thus provided with exit ventilators in the forecourts of the houses. . . . It might be thought that the result of the inspection of nearly 7,000 manholes yielded exceptional results, due to accumulation of stoppages long unrecognised. . . . I have, however, had a re-inspection made of about 500 manholes, with a view to seeing whether the results yielded by the first inspection were confirmed. The re-inspection was made within eight months of the first survey, and included 216 manholes where originally no defects were discovered, and 288 where defects originally found had been made good. In nearly 15 per cent. of the manholes re-examined, the most serious defects were discovered."

It must be admitted that the facts constitute a serious impeachment of the system, and should these results be confirmed by observations made over a wider area throughout the country, it is clear that our much-vaunted improvements in sanitary drainage will be sceptically viewed. The Departmental Committee, whatever its findings, has an important investigation to conduct, and the results of the inquiry will be awaited with keen interest by all who are concerned in the sanitary condition of dwelling houses.

